# Historical biogeography resolves the origins of endemic Arabian toad lineages (Anura: Bufonidae): Evidence for ancient vicariance and dispersal events with the Horn of Africa and South Asia

**DOI:** 10.1186/s12862-015-0417-y

**Published:** 2015-08-06

**Authors:** Daniel M. Portik, Theodore J. Papenfuss

**Affiliations:** Museum of Vertebrate Zoology and Department of Integrative Biology, 3101 Valley Life Sciences Building, University of California, Berkeley, CA 94720-3160 USA

**Keywords:** Arabian Peninsula, Historical biogeography, Amphibians, Toads, Red Sea

## Abstract

**Background:**

The Arabian Peninsula is home to a unique fauna that has assembled and evolved throughout the course of major geophysical events, including the separation of the Arabian Plate from Africa and subsequent collision with Eurasia. Opportunities for faunal exchanges with particular continents occurred in temporally distinct periods, and the presence of African, Western Eurasian, and South Asian derived taxa on the Arabian Peninsula signifies the complexity of these historical biogeographic events. The six true toad species (family Bufonidae) endemic to the Arabian Peninsula present a considerable taxonomic and biogeographic challenge because they are part of a global bufonid radiation, including several genera surrounding the Arabian Peninsula, and difficult to discriminate morphologically. As they could be derived from African, Western Eurasian, or South Asian toad groups, elucidating their evolutionary relationships has important implications for historical biogeography. Here, we analyze a global molecular data set of 243 bufonid lineages, with an emphasis on new sampling from the Horn of Africa, Western Eurasia, South Asia, and the Arabian Peninsula, to reconstruct the evolutionary relationships of the Arabian species. We produce a robust time-calibrated phylogeny to infer the biogeographic history of this group on and around the Arabian Peninsula.

**Results:**

Our phylogenetic analyses indicate two of the endemic Arabian toad species, “*Bufo*” *tihamicus* and “*Bufo*” *arabicus*, evolved independently within the African genus *Amietophrynus*. We confirm the Arabian species *Duttaphrynus dhufarensis* is of South Asian origin, but do not find evidence for the Asian genus *Duttaphrynus* being present in the Horn of Africa, discrediting a previously proposed Asian bufonid dispersal event to Africa. We also do not find evidence of the African genus *Amietophrynus* occurring in South Asia, suggesting that unlike many other vertebrate taxa, toads have not used the Arabian Peninsula as a stepping-stone for trans-continental dispersal. Our divergence dating estimates strongly suggest the formation of the Red Sea drove simultaneous divergences between two of the Arabian species (*A. tihamicus* comb. nov. and *A. arabicus* comb. nov.) and their closest mainland African relatives in the Early Miocene. We estimate the divergence of *D. dhufarensis* with its closest South Asian relatives occurred in the mid to Late Miocene, suggesting the temporary or permanent land connections between the Arabian plate and Eurasia facilitated dispersal of this lineage to the Arabian Peninsula.

**Conclusions:**

The Arabian bufonid assemblage, despite being comparatively depauperate with respect to surrounding continents, exemplifies the faunal pattern of the Arabian Peninsula, namely being a complex admixture of African, Western Eurasian, and South Asian elements. The historical biogeographic patterns exhibited by Arabian toads and their allies are concordant with studies of other vertebrate taxa, building support for the role of major geological events in driving simultaneous vicariance and dispersal events around the Arabian Peninsula. Although many taxa or groups exhibiting disjunct Afro-Arabian distributions appear to have dispersed more recently from the Horn of Africa via a southern land bridge or overwater dispersal, both *Amietophrynus tihamicus* and *A. arabicus* likely represent true African relicts resulting from vicariance associated with the Red Sea formation, a pattern that so far is rare among the vertebrate species investigated.

**Electronic supplementary material:**

The online version of this article (doi:10.1186/s12862-015-0417-y) contains supplementary material, which is available to authorized users.

## Background

**Fig. 1:**
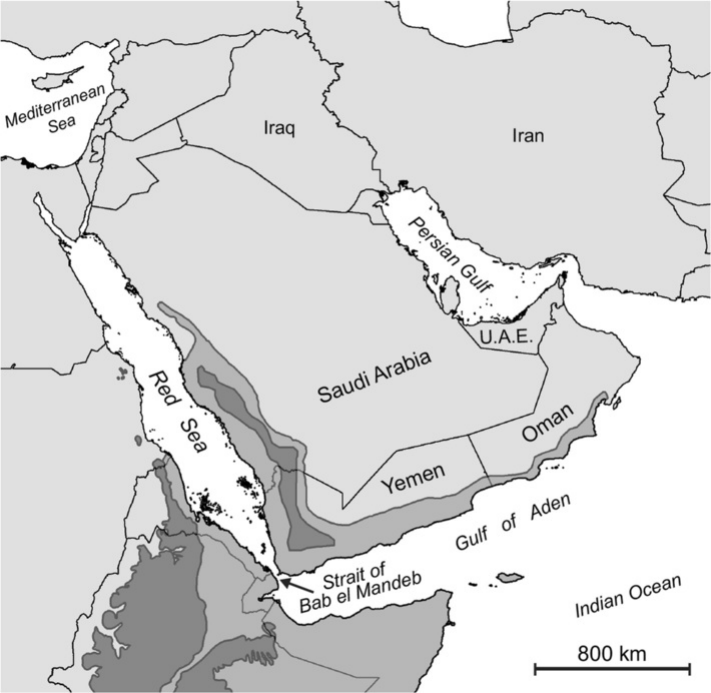
The Arabian Peninsula. A map of the Arabian Peninsula showing disjunct extensions of the Horn of Africa Hotspot (medium grey) and the Eastern Afromontane Hotspot (dark grey), along with pertinent seas and country borders. Shape files for hotspots were obtained through the “Biodiversity Hotspots” package produced by Conservation International (2011).

The Arabian Peninsula possesses a unique assemblage of plant and animal species that result from the dynamic geologic history and shifting climate of this region. The flora and fauna range from localized temperate endemics occurring in the disjunct extensions of the Horn of Africa Hotspot and the Eastern Afromontane Hotspot [1, 2] in the southwest regions (Fig. 1), to more broadly distributed arid-adapted clades occurring throughout much of the Arabian Peninsula [3–13]. Many large-scale biogeographic patterns for Arabian taxa have been profoundly shaped by the complex tectonic history of the Arabian Peninsula [14–22]. The tectonic activity ultimately responsible for the separation of the Arabian Plate from Africa began approximately 30 Ma, and rifting of the southern Red Sea occurred in the early Miocene (27 Ma), and a connection to the Neotethys Sea and was completed by 23 Ma [23, 24]. The formation of the Red Sea represented a major vicariance event for taxa with formerly continuous Afro-Arabian distributions. The isolated Arabian plate collided with the Anatolian plate approximately 18–16 Ma, forming the temporary *Gomphotherium* bridge [25, 26]. A more permanent land bridge was established after the Arabian plate collided with Eurasia approximately 15 Ma [24], which allowed regular faunal exchanges between North Africa, the Arabian Peninsula, and Eurasia. These land bridges also created opportunities for trans-continental dispersals using the Arabian Peninsula as a stepping-stone. Sea levels dropped throughout the Red Sea around 10 Ma, providing evidence for the closing of the Strait of Bab el Mandeb and establishment of a southern land bridge between Africa and Arabia [24]. Pliocene marine sediment deposits formed around 5.3 Ma, indicating this land bridge was subsequently lost, and although cyclical Pleistocene land bridges related to glacial cycles have been proposed [27], more recent evidence does not support their existence [28]. Conversely, there is evidence the Persian Gulf was reduced to a series of freshwater lakes [29] or completely waterless [30] during the late Pleistocene, providing a connection between the Arabian Peninsula and Southwest Asia. Plio-Pleistocene climate change is hypothesized to have driven speciation in the southwest montane regions of the Arabian Peninsula [3, 11], and is linked to aridification in the Neogene [31, 32]. This may explain additional biogeographic patterns, but on a much more localized scale.

The geologic activity associated with the Arabian Peninsula altered the historical connectivity of this landmass to surrounding continents, and as a result opportunities for faunal exchanges with particular continents occurred in temporally distinct periods. For example, taxa exhibiting disjunct Afro-Arabian distributions either exhibit early Miocene divergences associated with vicariance resulting from the Red Sea formation (30–23 Ma), or dispersed more recently from the Horn of Africa via a southern landbridge or overwater dispersal (10–5.3 Ma or 5.3 Ma to present, respectively) (reviewed in [18]). If the phylogenetic placement of an Arabian focal group is recovered, predictions can be made concerning the timing of speciation and tested in a temporally explicit phylogenetic framework to infer historical biogeographic patterns. The limited phylogenetic studies focusing on or including Arabian species have demonstrated concordant divergence timings for taxa with similar biogeographic origins [16–20], however these biogeographic patterns remain largely understudied for most Arabian taxa.

Although overall levels of endemism among vertebrate groups across the Arabian Peninsula are moderate, Arabian amphibians exhibit a high degree of endemism [15, 33]. Of the nine described Arabian anuran species, six of these species are true toads belonging to the family Bufonidae, a clade that contains close to 600 described species and which exhibits a nearly worldwide distribution [34]. The dispersal ability of toads is limited by their reliance on freshwater habitat for breeding and their intolerance to saltwater, making them an interesting system for investigating historical biogeography in this region. Several broad-scale phylogenetic studies of bufonids have revealed the existence of largely discrete continental clades [35–39] that are now accepted as distinct genera or subgenera [34, 40]. Three such genera are currently distributed in geographic regions directly surrounding the Arabian Peninsula, including *Amietophrynus* (Africa), *Bufotes* (Northern Eurasia and North Africa), and *Duttaphrynus* (Southwest and South Asia), allowing the possibility of multiple biogeographic origins for the Arabian species.

Due to a lack of molecular sampling, the assignment of the Arabian bufonid species to genera, and therefore biogeographic origin, has long been problematic. One Arabian toad species, *Bufotes* cf. *variabilis*, is attributable to the Western Eurasian *Bufotes viridis* species complex on the basis of morphology [33, 41]. However, the affinities of the other Arabian toads are unresolved in part due to the similar morphology among the many species of toads occurring in regions surrounding the Arabian Peninsula. Based on molecular evidence the Arabian endemic *Duttaphrynus dhufarensis* was recently determined to be of Asian origin, with its closest relatives occurring on the Indian subcontinent [37], although several taxa occurring across Southwest Asia were not included in the analysis. This discovery, and the assignment of *D. dodsoni* (a species distributed throughout the Horn of Africa) to the genus [37, 40], implies the Arabian Peninsula has acted as a stepping-stone for Asian-derived bufonid species to colonize mainland Africa. This scenario remains to be tested in a phylogenetic framework, but has important biogeographic implications. Other than *B*. cf. *variabilis* and *D. dhufarensis*, the remaining Arabian toads could not be allocated to genera, and remain in a non-taxon *Bufo* that is currently polyphyletic and is denoted as “*Bufo*”. These species include *“Bufo” arabicus, “Bufo” tihamicus, “Bufo” hadramautinus*, and *“Bufo” scorteccii*. Both *“Bufo” arabicus* and *D. dhufarensis* possess the largest geographic distribution of the Arabian toads, whereas *“Bufo” tihamicus* is only distributed along the coastal Red Sea region. Although *“Bufo” tihamicus* is thought to be a close relative of the Sahelian distributed African taxon *“Bufo” pentoni*, the latter species is also currently not assigned to a genus, and therefore the phylogenetic relationships of this complex remains convoluted. The species *“Bufo” hadramautinus* and *scorteccii* are restricted to one or two localities, and their validity has been questioned as they may represent isolated phenotypically variable populations of the wider ranging *“Bufo” arabicus* and *D. dhufarensis*, respectively [33]. As a result of these hypotheses “*Bufo*” *scorteccii* has been tentatively assigned to the genus *Duttaphrynus* [40], although this taxonomic assignment is certainly premature, and some authors have regarded *“Bufo” hadramautinus* as a synonym of *“Bufo” arabicus* [42].

The evolution of Arabian bufonids and their close relatives remains a challenging biogeographic and taxonomic problem, and it is unknown if the unassigned Arabian toads are derived from African, Southwest Asian, or Western Eurasian lineages, or are the result of in-situ diversification on the Peninsula. In addition, the biogeographic patterns of the genus *Duttaphrynus* have not been investigated yet have important implications concerning whether amphibians successfully colonized the Horn of Africa using the Arabian Peninsula as a stepping-stone. A similar problem persists for *“Bufo” tihamicus* and *pentoni*, which could be derived from African, Southwest Asian, or Western Eurasian clades, each of which has implies different biogeographic scenarios for explaining the current geographic distributions of these two species.

Through several years of fieldwork, TJP obtained key samples of Arabian taxa (including *“Bufo” arabicus, “Bufo” tihamicus, D. dhufarensis*) as well as many biogeographically important species surrounding the Arabian Peninsula (including “*Bufo*” *pentoni* and *D. dodsoni*), finally allowing an assessment of their evolutionary relationships using a molecular phylogenetic framework. We seek to untangle the various biogeographic scenarios resulting in the evolution of the Arabian bufonids and their close relatives. We aim to identify the closest relatives of *“Bufo” tihamicus* and *“Bufo” arabicus* to determine if they are derived from African, Western Eurasian, or South Asian lineages. If they are African in origin, we predict they will have diverged from their closest mainland relatives either 1) following the formation of the Red Sea (23 Ma), or 2) as a result of dispersal across a southern Afro-Arabian land bridge (10–5.3 Ma). Alternatively, if they are Western Eurasian or South Asian in origin, we predict they will have colonized the Arabian Peninsula following establishment of Eurasian land bridge connections (18–15 Ma). We also seek to test the stepping-stone colonization of the Horn of Africa by the genus *Duttaphrynus*, and predict a sister-taxon relationship between *D. dodsoni* and *D. dhufarensis,* with a divergence time following permanent Eurasian land bridge connections (15 Ma). We use newly generated multi-locus molecular data and a comprehensive data set of published bufonid sequences to reconstruct phylogenetic relationships to identify the origins of the Arabian taxa. We use these molecular data to estimate divergence times to identify key biogeographic events that could have led to the formation of these lineages. We examine our results in the context of prior studies that have investigated historical biogeographic patterns in other Arabian taxa, and highlight areas requiring further study.

## Results

### Phylogenetic relationships

**Fig. 2:**
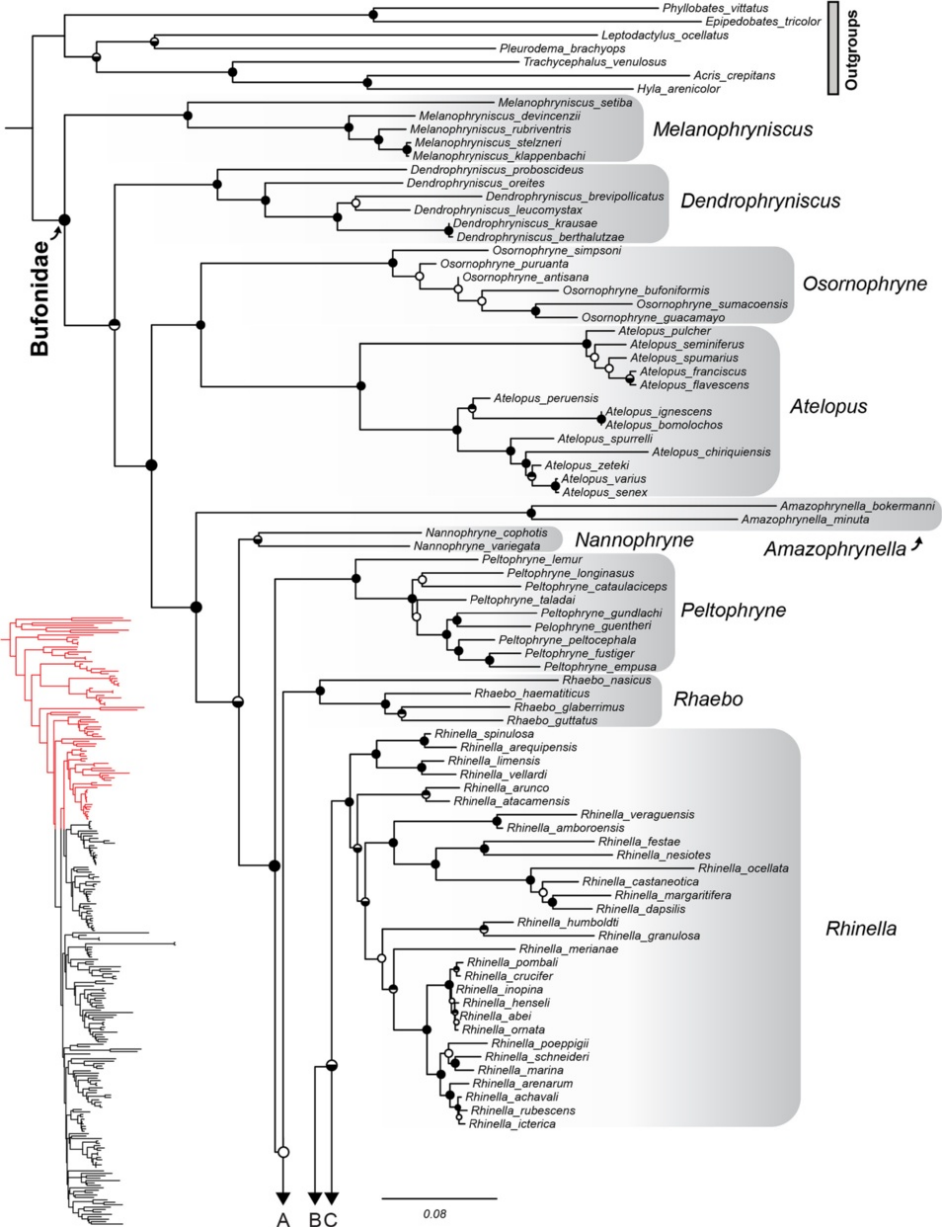
Phylogenetic relationships of global bufonids, part one. The maximum likelihood tree of the Bufonidae, based on 13 loci and 243 taxa. Filled circles on nodes represent high support (Bayesian posterior probabilities [BPP] > 0.95 and maximum likelihood bootstrap scores [MLBS] > 70%); circles with top half fill: MLBS > 70% and BPP < 0.95; circles with bottom half fill: MLBS < 70% and BPP > 0.95; open circles represent support values less than given threshold for both analysis types. Branch lengths are proportional to substitutions/site, indicated by scale bar below, and genera are outlined in grey shading. Letters below correspond to connections to Fig 3. The portion of the phylogeny represented is highlighted in red on the full topology.

**Fig. 3:**
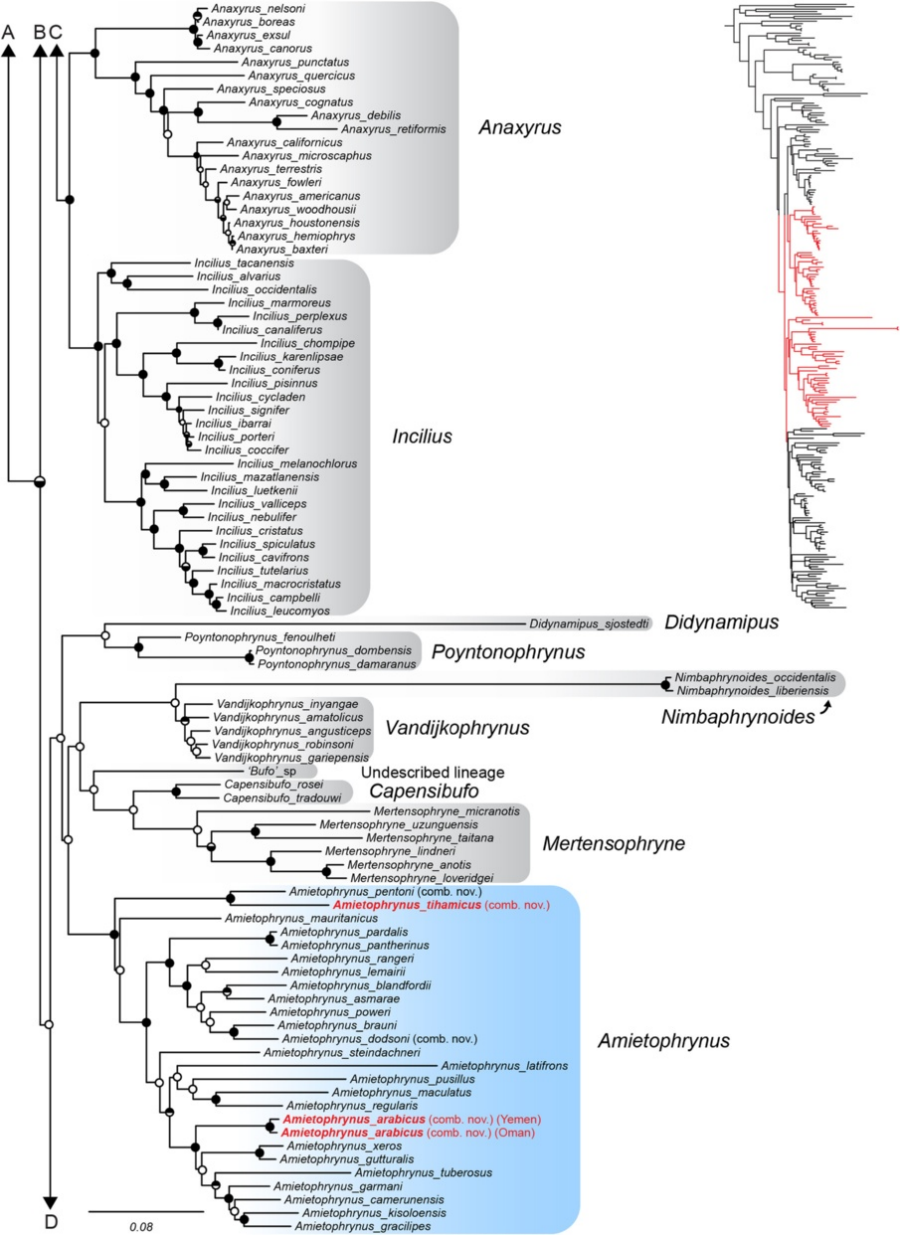
Phylogenetic relationships of global bufonids, part two. The maximum likelihood tree of the Bufonidae, based on 13 loci and 243 taxa. Filled circles on nodes represent high support (BPP > 0.95 and MLBS > 70%); circles with top half fill: MLBS > 70% and BPP < 0.95; circles with bottom half fill: MLBS < 70% and BPP > 0.95; open circles represent support values less than given threshold for both analysis types. Branch lengths are proportional to substitutions/site, indicated by scale bar below. Genera are outlined in grey shading, genera containing Arabian taxa are colored and Arabian species are denoted by red bold text. Letters above correspond to connections to Fig. 2, and below to Fig. 4. The portion of the phylogeny represented is highlighted in red on the full topology.

**Fig. 4:**
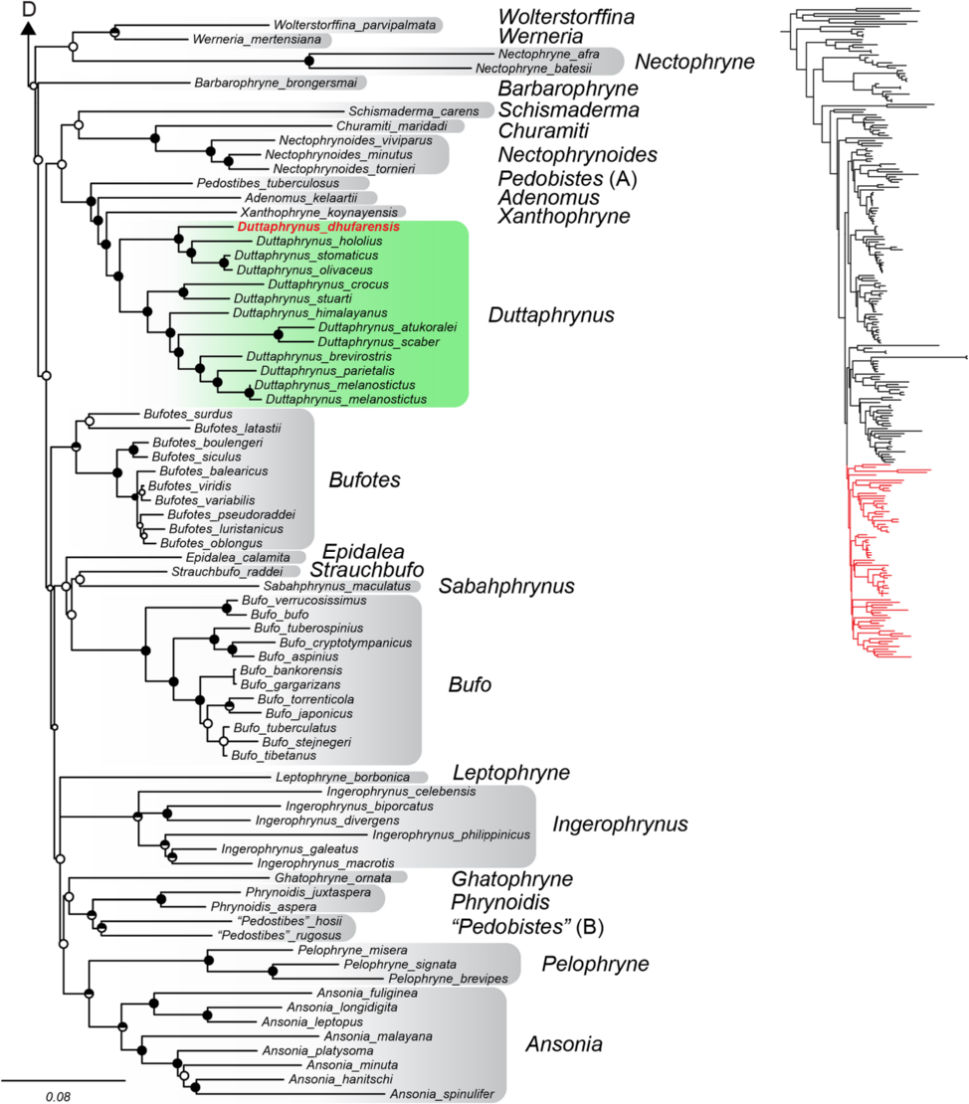
Phylogenetic relationships of global bufonids, part three. The maximum likelihood tree of the Bufonidae, based on 13 loci and 243 taxa. Filled circles on nodes represent high support (BPP > 0.95 and MLBS > 70%); circles with top half fill: MLBS > 70% and BPP < 0.95; circles with bottom half fill: MLBS < 70% and BPP > 0.95; open circles represent support values less than given threshold for both analysis types. Branch lengths are proportional to substitutions/site, indicated by scale bar below. Genera are outlined in grey shading, genera containing Arabian taxa are colored and Arabian species are denoted by red bold text. Letters above correspond to connections to Fig. 3. The portion of the phylogeny represented is highlighted in red on the full topology.

The overall phylogenetic relationships within the family Bufonidae are concordant across analyses and are presented in Figs. 2-4 (full length figure available in Additional file 1). We find strong support for the monophyly of the Bufonidae, and relationships among the basal genera (*Melanophryniscus, Dendrophryniscus, Osornophryne, Atelopus, Amazophrynella, Nannophryne, Peltophryne*) are well resolved and consistent with previous studies [35–39]. Our phylogenetic analyses place *Anaxyrus* as the sister clade to *Incilius* with high support, and together these two genera are moderately supported as being sister to the genus *Rhinella* (Figs. 2, 3). Beyond this grouping, the relationships among the other derived genera (*Rhaebo, Didynamipus, Poyntonophrynus, Nimbaphrynoides, Vandijkophrynus, Capensibufo, Mertensophryne, Amietophrynus, Wolterstorffina, Werneria, Nectophryne, Barbarophryne, Schismaderma, Churamiti, Nectophrynoides, Pedobistes, Adenomus, Xanthophryne, Duttaphrynus, Bufotes, Epidalea, Strauchbufo, Sabahphrynus, Bufo, Leptophryne, Ingerophrynus, Ghatophryne, Phrynoidis, Pelophryne, Ansonia*) are largely unresolved (Figs. 3, 4). Genera sampled for more than one lineage are supported as monophyletic, with the exception of *Pedobistes* (as found by Van Bocxlaer et al. [38]). This is true for the speciose genera surrounding the Arabian Peninsula, including *Amietophrynus*, *Bufotes*, and *Duttaphrynus*, which are all recovered as independent monophyletic groups with strong support (Figs. 3, 4). In this global bufonid species data set with an emphasis on sampling surrounding the Arabian Peninsula, our analyses consistently place the Arabian toad species within particular genera.

**Fig. 5:**
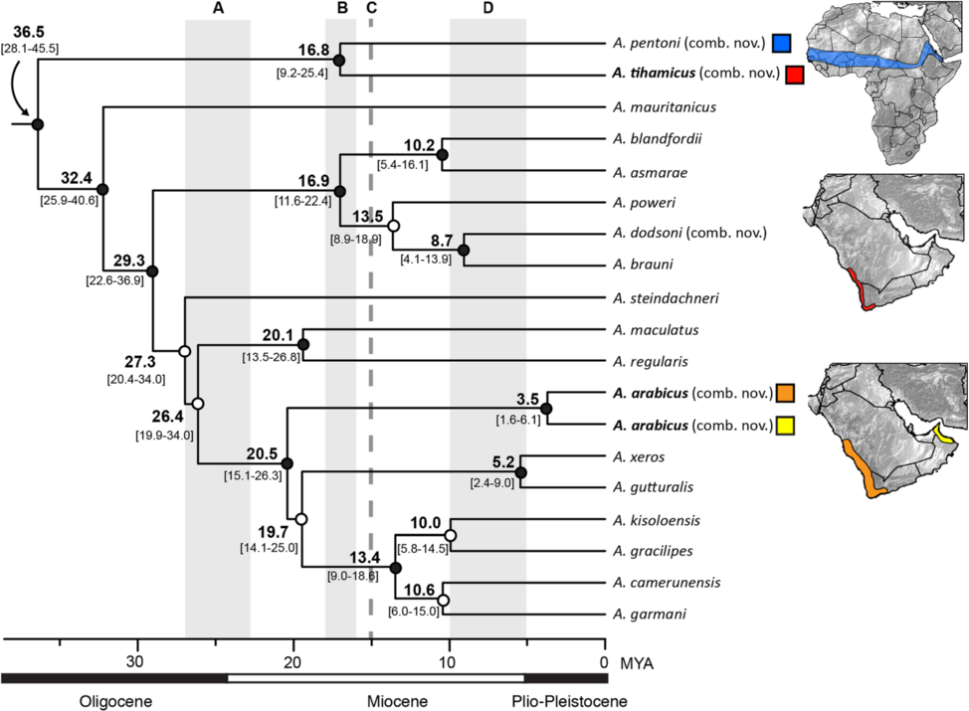
Divergence timings of the genus *Amietophrynus.* Bayesian maximum clade credibility chronogram of the genus *Amietophrynus* inferred in BEAST (Analysis 2) with median divergence times and associated 95 % highest posterior distributions of dates in brackets. Timings of relevant geologic events are illustrated: A – Red Sea formation (27–23 Ma); B – *Gomphotherium* bridge (18–16 Ma); C – permanent Eurasian landbridge (15 Ma); D – southern Afro-Arabian landbridge (10–5.3 Ma). Ranges of Arabian species and, if relevant, corresponding sister taxon are illustrated. Filled circles on nodes represent Bayesian posterior probabilities > 0.95, open circles represent support values < 0.95.

**Fig. 6:**
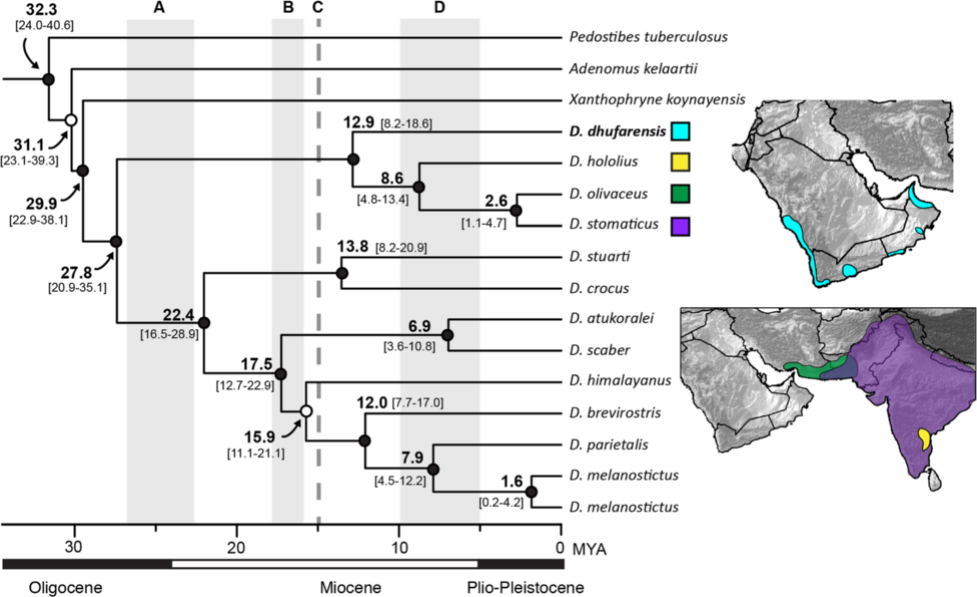
Divergence timings of the genus *Duttaphrynus.* Bayesian maximum clade credibility chronogram of the genus *Duttaphrynus* inferred in BEAST (Analysis 2) with median divergence times and associated 95 % highest posterior distributions of dates in brackets. Timings of relevant geologic events are illustrated: A – Red Sea formation (27–23 Ma); B - *Gomphotherium* bridge (18–16 Ma); C – permanent Eurasian landbridge (15 Ma); D – southern Afro-Arabian landbridge (10–5.3 Ma). Ranges of Arabian species and, if relevant, corresponding sister taxon are illustrated. Filled circles on nodes represent Bayesian posterior probabilities > 0.95, open circles represent support values < 0.95.

The Arabian species “*Bufo*” *tihamicus* is strongly supported as the sister taxon of the African Sahelian distributed “*Bufo*” *pentoni*, and together these two lineages are recovered as sister to all other species of *Amietophrynus* (Figs. 3, 5). Our analyses recover “*Bufo*” *arabicus* as the sister taxon to a grouping of two clades. One clade consists of the more arid-adapted species *A. xeros* and *A. gutturalis*, and the other consists of *A. tuberosus*, *A. garmani*, *A. camerunensis*, *A. kisolensis*, and *A. gracilipes*. The resolution of the relationship between “*Bufo*” *arabicus* and these two clades is not strongly supported, with some results placing “*Bufo*” *arabicus* as sister to *A. xeros* and *A. gutturalis,* and others placing “*Bufo*” *arabicus* as sister to both clades (as in Fig. 3). Our results confirm the findings of Van Bocxlaer et al. [37] in recovering the Arabian species *Duttaphrynus dhufarensis* within the Asian genus *Duttaphrynus*. Our improved sampling from Iran and Pakistan places *D. dhufarensis* as sister to a clade consisting of *D. hololius*, *D. stomaticus* and *D. olivaceus* with strong support (Figs. 4, 6). *Duttaphrynus dodsoni*, which is distributed across the Horn of Africa, is not recovered as a member of the genus *Duttaphrynus*, but rather is found deeply nested in the genus *Amietophrynus* and is a close relative of the East African species *Amietophrynus brauni* (Figs. 3, 5).

In addition to elucidating the relationships of Arabian bufonids, our phylogenetic analyses revealed a unique bufonid lineage restricted to the Horn of Africa, designated as “*Bufo*” sp. (Fig. 3, “Undescribed lineage”). The placement of this lineage is not well resolved and it does not appear to be a member of any currently described African genus (Figs. 2-4). Maximum likelihood and Bayesian consensus trees show this new genus is most closely related to a clade of African genera (*Capensibufo*, *Mertensophryne, Vandijkophrynus*, and *Nimbaphrynoides*) but this relationship is not strongly supported (Fig. 3). An additional extensive comparison with over 500 unpublished 16S sequences of mainly African bufonids also failed to resolve the placement of this taxon with any described genus (H.C. Liedtke, pers. comm.), and additional taxonomic work is therefore required for this unique lineage.

### Divergence dating estimates

The three divergence dating analyses (A1, A2, A3) produced largely congruent phylogenetic topologies (Additional file 5). Divergence dating estimates varied little between analyses, though the 95 % highest posterior density regions are slightly broader in analyses A1 and A3 than those in A2 (Table 1, Additional file 6). All dating analyses suggest a mid-Cretaceous origin of the family Bufonidae, with median estimates ranging between 93.5–100.3 Ma ([A1: 94.9 Ma, 95 % HPD 64.6–129.8]; [A2: 93.5, 73.0–114.0 Ma]; [A3: 100.3, 70.1–133.1 Ma]. These dates are consistent with Pramuk et al. [36], who also recover a mid-Cretaceous origin of the family with an estimate of 88.2 Ma (78.3–98.8 Ma), but are approximately 20–25 million years older than dates recovered by Van Bocxlaer et al. [38] for the group (67.9 Ma, 95 % HPD 52.7–92.7]. Similar to both Pramuk et al. [36] and Van Bocxlaer et al. [38], we find the origin and diversification of most derived (eg. formerly *Bufo*) bufonid genera occurred in a window of approximately 10 million years. Our estimates place this rapid diversification in the Eocene, as found by Pramuk et al. [36], though Van Bocxlaer et al. [38] date this radiation to the Oligocene.

**Table 1:** Key results of divergence dating analyses

	Dating analysis		
Node label	A1	A2	A3
Root age	124.8 [84.1–177.1]	121.5 [89.3–160.3]	128.0 [85.7–182.1]
Bufonidae	94.9 [64.6–129.8]	93.5 [73.0–114.0]	100.3 [70.1–133.1]
Origin *Amietophrynus*	37.6 [25.6–51.5]	36.5 [28.1–45.5]	37.2 [27.1–50.4]
TMRCA *A. arabicus*	21.2 [14.2–29.2]	20.5 [15.1–26.3]	20.7 [14.8–29.2]
TMRCA *A. tihamicus*	17.9 [9.0–26.6]	16.8 [9.3–25.4]	17.0 [9.3–25.9]
Origin *Duttaphrynus*	29.3 [19.3–40.5]	27.8 [20.9–35.1]	29.2 [20.4–40.0]
TMRCA *D. dhufarensis*	13.5 [7.9–20.9]	12.9 [8.2–18.6]	13.5 [8.4–20.1]

For relevant nodes, median age and 95 % highest posterior density region are given (Ma). Analysis 1 (A1): inclusion of four internal calibrations with lognormal calibration prior for age of the Bufonidae; Analysis 2 (A2): inclusion of four internal calibrations with normal calibration prior for age of the Bufonidae; Analysis 3 (A3): inclusion of four internal calibrations with exponential calibration prior for age of the Bufonidae.

Based on our data, diversification of the genus *Amietophrynus* began around the Eocene-Oligocene boundary ([A1: 37.6, 25.6–51.5 Ma]; [A2: 36.5, 28.1–45.5 Ma]; [A3: 37.2, 27.1–50.4 Ma]) whereas comparatively diversification began more recently in *Duttaphrynus* during the mid-Oligocene ([A1: 29.3, 19.3–40.5 Ma]; [A2: 27.8, 20.9–35.1 Ma]; [A3: 29.2, 20.4–40.0 Ma]) (Table 1). The divergences of the Arabian species (*“Bufo” tihamicus* and *arabicus*, *D. dhufarensis*) are estimated to have occurred during the Miocene. An Early Miocene divergence is estimated between *“Bufo” tihamicus* and *“Bufo” pentoni* ([A1: 17.9, 9.0–26.6 Ma]; [A2: 16.8, 9.2–25.4 Ma]; [A3: 17.0, 9.3–25.9 Ma]) as well as the split between *“Bufo” arabicus* and its sister clade ([A1: 21.2, 14.2–29.2 Ma]; [A2: 20.5, 15.1–26.3 Ma]; [A3: 20.7, 14.8–29.2 Ma]) (Fig. 5, Table 1). Within *“Bufo” arabicus*, the allopatric populations occurring in Yemen and Oman are supported as genetically distinct and are estimated to have diverged in the Pliocene ([A1: 4.0, 1.5–6.7 Ma]; [A2: 3.5, 1.6–6.1 Ma]; [A3: 3.5, 1.5–7.3 Ma]) (Fig. 5). The divergence estimates for the split between *Duttaphrynus dhufarensis* and the clade consisting of *D. hololius*, *D. stomaticus* and *D. olivaceus* occur in the mid-Miocene, with median estimates ranging from 12.9–13.5 Ma ([A1: 13.5, 7.9–20.9 Ma]; [A2: 12.9, 8.2–18.6 Ma]; [A3: 13.5, 8.4–20.1 Ma]) (Fig. 6). These dates are slightly older than those recovered by Van Bocxlaer et al. [37], who estimate this divergence at 8.5 Ma (95 % HPD 5.9–12.3). Previously untested, the divergence event between closely related *D. stomaticus* and *D. olivaceus* is estimated to have occurred in the late Pliocene ([A1: 3.0, 1.0–5.1 Ma]; [A2: 2.6, 1.1–4.7 Ma]; [A3: 2.7, 1.0–5.2 Ma]; Fig. 5).

## Discussion

### Evolutionary relationships of Arabian bufonids

The relationships among Arabian toad species have long been problematic, owing in part to the generalized morphology of these species and of lineages in surrounding regions [33, 45]. The molecular delimitation of at least three speciose toad genera differentially distributed around the Arabian Peninsula (*Amietophrynus, Bufotes, Duttaphrynus*) further highlighted the possibility of several alternative biogeographic origins for the Arabian species [35]. Prior to our study, the Arabian bufonid species assemblage was recognized as having both Western Eurasian and Asian elements (*Bufotes* cf. *variabilis* [33, 41], *Duttaphrynus dhufarensis* [37]), and here we identify a previously unrecognized, though not unexpected, African component. We find strong support for a sister relationship between “*Bufo*” *pentoni* and “*Bufo*” *tihamicus*, which together form a monophyletic assemblage with all other species in the African genus *Amietophrynus* (Figs. 3, 5). The Arabian lineage “*Bufo*” *arabicus* is also recovered in this clade (Figs. 3, 5), and our results do not support a close relationship between these Arabian lineages. Rather, these species have independent evolutionary origins in the genus.

We recover the Arabian species *Duttaphrynus dhufarensis* as part of a largely South Asian clade, represented by other species in the genus *Duttaphrynus* (Figs. 4, 6), consistent with Van Bocxlaer et al. [37]. In their study, Van Bocxlaer et al. [37] recover *D. dhufarensis* as sister to a clade containing *D. stomaticus* and *D. hololius*. They report *D. hololius* as widespread on the Indian subcontinent, with *D. stomaticus* being restricted to the Western Ghats [37:Fig. 2]. The true distributions of these species are actually converse, though their interpretation of the biogeographic results is sound. Regardless, both species were sampled from India, and together with the other species included in their analyses the westernmost sampling for the genus was limited to India. This left a vast sampling gap across Pakistan and Iran, a region that contains many widespread species in the genus *Duttaphrynus* and *Bufotes*. Our sampling includes six species from this region (*Bufotes oblongus, B. pseudoraddei*, *B. surdus, B. variabilis*, *D. olivaceus*, and *D. stomaticus*) and includes geographically relevant localities adjacent to the Gulf of Oman and Persian Gulf in Iran and Pakistan. With this biogeographically improved sampling, we find *D. dhufarensis* is the sister taxon to a clade containing *D. hololius*, *D. olivaceus* and *D. stomaticus*. We find *D. stomaticus* exhibits a distribution throughout Pakistan and India, and the *D. olivaceus* is distributed largely throughout Iran and into Pakistan (Figs. 4, 6).

With our broad species sampling of *Duttaphrynus*, *Bufotes*, and *Amietophrynus*, our analyses do not recover *Duttaphrynus dodsoni* as monophyletic with other members of the genus. Instead, this species is strongly supported as nested within the African distributed genus *Amietophrynus* as sister taxon to the East African species *Amietophrynus brauni* (Figs. 3, 5). We discuss the biogeographic implications of this discovery and the overall historical biogeography of the Arabian toad species below.

### Historical biogeography of Arabian toads

Our phylogenetic analyses have resolved the origins of several biogeographically interesting toad lineages distributed throughout the Horn of Africa, the Arabian Peninsula, and Southwest Asia, and here we interpret major divergence events in the context of the geological history of this region.

The species *“Bufo” arabicus* and *“Bufo” tihamicus* are both estimated to have diverged from their closest African relatives in the Early Miocene, approximately 20.5–21.2 Ma (95 % HPD range: 14.2–29.2 Ma) and 16.8–17.9 Ma (95 % HPD range: 9.0–26.6 Ma), respectively (Fig. 5, Table 1). These timings are remarkably concordant considering these two lineages have evolved independently within the genus. The age estimates are quite ancient and rule out dispersal across a southern Afro-Arabian landbridge (10–5.3 Ma) and overwater dispersal (5.3 Ma to present), but are consistent with the separation of the Arabian Plate from mainland Africa as a result of the Red Sea formation during the Oligocene-Miocene boundary (27–23 Ma) (Fig. 5) [24, 25]. The landbridge spanning the Strait of Bab el Mandeb, which created a dispersal route between Arabian Peninsula and the Horn of Africa, was not established until 10–5.3 Ma. Many of the African-derived terrestrial vertebrate taxa present in the southwestern Arabian Peninsula arrived by dispersing from the Horn of Africa beginning in the late Miocene via a southern land bridge or overwater dispersal events [17, 18, 20, 22] rather than originating as a result of the initial separation of the Arabian plate from Africa. This classifies the Arabian toad species *“Bufo” arabicus* and *“Bufo” tihamicus* as true African relicts, a pattern that has only been demonstrated for one other taxon, the viper species *Echis coloratus*, which also diverged in the Early Miocene [17].

The Asian derived species *Duttaphrynus dhufarensis* is estimated to have diverged in the mid-Miocene, 12.9–13.5 Ma (95 % HPD range: 7.9–20.9 Ma) (Fig. 6, Table 1). The temporary connections between the Arabian plate and Eurasia established 18–16 Ma are thought to have allowed the first faunal exchanges between these distinct biogeographic regions, with a permanent Eurasian connection being established ~15 Ma [24–26]. The divergence estimates for *D. dhufarensis* are congruent with the temporal range of these events, and the establishment of land bridges likely served as dispersal routes for this lineage to colonize the Arabian Peninsula.

Following a stepping stone model of dispersal, if the genus *Duttaphrynus* occurred on mainland Africa, the temporal origins of these lineages would be expected to be younger than the age of *D. dhufarensis* (13 Ma; 7.9–20.9 Ma). However, with the discovery of the phylogenetic placement of *Duttaphrynus dodsoni* in the genus *Amietophrynus*, we find no support for the genus occurring on the Horn of Africa, indicating South Asian-derived bufonid lineages did not successfully complete trans-continental dispersals across the Arabian Peninsula. Additionally, based on our extensive sampling of the Horn of Africa, Arabian Peninsula, and South Asia, we find no evidence for the stepping-stone model of dispersal across the Arabian Peninsula for any African (*Amietophrynus*) or Asian (*Duttaphrynus*) bufonid species (Figs. 2-6), as no species of *Amietophrynus* are found in Iran or Pakistan. This is somewhat unexpected because this model has been invoked to explain ancient Asiatic-African dispersals in multiple groups of ranoid frogs [43]. Additionally, there is evidence suggesting the Persian Gulf region was greatly reduced or dry in the Pleistocene [29, 30]. The apparent lack of dispersals of toad lineages across the Persian Gulf region during this time period is surprising, as population exchanges of the viper species *Echis carinatus* likely occurred through this route [17].

### Taxonomic implications

Based on our sampling strategy and the phylogenetic placement of key species, we recommend several taxonomic changes. The species “*Bufo*” *tihamicus* and “*Bufo*” *pentoni* form a monophyletic group with all other *Amietophrynus*, and although they represent the most basal divergence in the group we recommend assignment to the genus, and recognize *Amietophrynus tihamicus* comb. nov. and *Amietophrynus pentoni* comb. nov. The assignment of these two species can be further tested through karyotyping, as the 20-chromosome condition is considered apomorphic for this group [44, 45]. If an alternative chromosome condition is discovered in these two species, additional insight into chromosome evolution among bufonids will be gained and their generic assignment can be reconsidered. The Arabian species “*Bufo*” *arabicus* can be confidently assigned to the genus *Amietophrynus*, as *Amietophrynus arabicus* comb. nov., and *Duttaphrynus dodsoni* is also transferred to the genus as *Amietophrynus dodsoni* comb. nov.

The origins of several Arabian bufonids have been investigated, however several lineages require further investigation. The Arabian population of *Bufotes* cf. *variabilis* warrants further study to determine its distinctiveness with respect to *B. variabilis* sensu stricto. This population shares a similar distribution with *Hyla felixarabica*, a recently described Arabian species that was previously thought to be an isolate of a wider ranging Western Eurasian species complex (*Hyla arborea*) [15]. Additionally, the relationships of *“Bufo” hadramautinus* and *“Bufo” scorteccii* remain speculative. Although *“Bufo” hadramautinus* is morphologically similar to *Amietophrynus arabicus* [33], *“Bufo” scorteccii* is morphologically intermediate between *A. arabicus* and *D. dhufarensis* [33]. On this basis, *“Bufo” hadramautinus* can be tentatively recognized as *Amietophrynus hadramautinus* comb. nov., but *“Bufo” scorteccii* remains problematic and should remain unassigned until further study. Although inconvenient, there are major biogeographic implications associated with assignment to particular genera, and this action would circumvent such issues. When population-level sampling becomes available, additional phylogenetic work can clarify if these two geographically restricted lineages are: 1) intraspecific populations of one of the widespread Arabian bufonid species, 2) distinct lineages derived independently from surrounding continental faunas, or 3) distinct lineages resulting from in-situ speciation of an Arabian species, and further taxonomic assessments can be made.

## Conclusions

The Arabian Peninsula is home to a unique fauna that has assembled and evolved throughout the course of major geophysical events. The Arabian species assemblage represents an admixture of African, Western Eurasian, and South Asian elements, and this pattern is exemplified even in the relatively depauperate Arabian bufonids. In addition to having South Asian and Western Eurasian lineages, we have identified two lineages independently derived from continental Africa. Our dating estimates strongly suggest *Amietophrynus arabicus* and *A. tihamicus* did not colonize the Arabian Peninsula through overwater dispersal or a southern land bridge from the Horn of Africa, rather the formation of the Red Sea likely drove simultaneous divergences in these species. In this sense, they represent true African relicts in their current distribution on the Arabian Peninsula. More importantly, across all dating analyses the relative timing of divergence for these species is considerably older than that for the South Asian derived *Duttaphrynus dhufarensis*. These results conform to predictions based on geological events that species dispersing to the Arabian Peninsula across Eurasian land bridges should be younger in origin than true African relicts. Our investigation has revealed the stepping-stone hypothesis for trans-continental Afro-Asian bufonid dispersals is not accurate, and we find no evidence for *Amietophrynus* or *Duttaphrynus* species distributed outside of their main continental range and the Arabian Peninsula. Further studies of the remaining Arabian amphibian species can test if these biogeographic scenarios hold true for not only bufonids but also other anuran families. These amphibian studies and comparative studies of other terrestrial vertebrates can provide a clearer picture of the diversification of the unique faunal assemblage present on the Arabian Peninsula.

## Methods

### Taxon sampling

Due to the uncertainty of the phylogenetic placement of Arabian bufonid taxa, we sampled broadly across the family Bufonidae with an emphasis on regions directly surrounding the Arabian Peninsula (Table 2). We generated new multi-locus sequence data for 114 bufonid samples from 21 recognized species distributed across the Horn of Africa, the Arabian Peninsula, and Southwest Asia (Table 2, Additional file 2).

**Table 2:** New sampling of taxa included for this study

Species	Museum no.	Latitude	Longitude	Country
Amietophrynus arabicus*	CAS 250888	25.2651	56.3068	Oman
	**CAS 250889**	25.2651	56.3068	Oman
	CAS 250890	25.2651	56.3068	Oman
	CAS 250907	25.387	56.2649	Oman
	CAS 250908	25.387	56.2649	Oman
	CAS 251024	22.6052	59.0886	Oman
	CAS 251026	22.6052	59.0886	Oman
	CAS 251027	22.6052	59.0886	Oman
	CAS 251126	20.6888	58.2949	Oman
	CAS 251127	20.6888	58.2949	Oman
	CAS 251130	20.6888	58.2949	Oman
	CAS 251147	23.0548	57.4679	Oman
	CAS 251148	23.0548	57.4679	Oman
	CAS 251149	23.0548	57.4679	Oman
	CAS 251166	23.0713	57.6042	Oman
	CAS 251167	23.0713	57.6042	Oman
	MVZ 236403	15.3443	44.217	Yemen
	**MVZ 236407**	15.4693	44.2618	Yemen
	MVZ 236866	24.2633	56.1633	Oman
	MVZ 241304	23.0525	57.4691	Oman
	MVZ 241305	23.0525	57.4691	Oman
	MVZ 241306	23.0713	57.6043	Oman
	MVZ 241307	23.0713	57.6043	Oman
*Amietophrynus asmarae*	MVZ 257839			Ethiopia
	MVZ 257840			Ethiopia
	MVZ 257843			Ethiopia
	MVZ 257844			Ethiopia
	MVZ 257847			Ethiopia
	**MVZ 257848**			Ethiopia
	MVZ 257849			Ethiopia
	MVZ 257850			Ethiopia
	MVZ 257852			Ethiopia
*Amietophrynus blandfordii*	MVZ 241309	9.9493	43.2193	Somalia
	MVZ 241313	9.9698	43.4325	Somalia
	MVZ 241314	9.9698	43.4325	Somalia
	MVZ 241316	9.9698	43.4325	Somalia
	MVZ 241317	9.9698	43.4325	Somalia
	MVZ 241318	9.9698	43.4325	Somalia
	**MVZ 242725**	11.0261	49.193	Somalia
Amietophrynus dodsoni*	MVZ 241310	9.9493	43.2193	Somalia
	**MVZ 241312**	9.9493	43.2193	Somalia
*Amietophrynus garmani*	**MVZ 257841**			Ethiopia
	MVZ 257846			Ethiopia
*Amietophrynus mauritanicus*	MVZ 235679	36.9641	8.8822	Tunisia
Amietophrynus pentoni*	MVZ 235732	16.5796	−15.8735	Mauritania
	**MVZ 249297**	9.24191	−1.84415	Ghana
	MVZ 249298	9.24191	−1.84415	Ghana
*Amietophrynus regularis*	MVZ 235735	16.5165	−15.8135	Mauritania
	MVZ 238858	13.5036	2.1135	Niger
	MVZ 238859	13.5036	2.1135	Niger
	MVZ 249302	9.259	−1.8541	Ghana
*Amietophrynus steindachneri*	MVZ 234101	−3.1999	40.0077	Kenya
	MVZ 234102	−3.1999	40.0077	Kenya
Amietophrynus tihamicus*	**MVZ 236409**	14.8238	43.1273	Yemen
	MVZ 236413	14.8238	43.1273	Yemen
*Amietophrynus xeros*	MVZ 235737	16.5165	−15.8135	Mauritania
	MVZ 238867	17.3868	7.9563	Niger
	MVZ 238868	17.3868	7.9563	Niger
*“Bufo”* sp.	**MVZ 242731**	11.2493	49.2678	Somalia
	MVZ 242732	11.2493	49.2678	Somalia
	MVZ 242733	11.2493	49.2678	Somalia
	MVZ 242776	11.0261	49.193	Somalia
*Bufotes oblongus*	MVZ 241548	36.2797	60.548	Iran
	MVZ 245904	32.8198	59.2171	Iran
	MVZ 245905	32.8198	59.2171	Iran
	MVZ 245906	32.8198	59.2171	Iran
	MVZ 245910	33.631	57.1616	Iran
	MVZ 245911	33.631	57.1616	Iran
	MVZ 245912	33.631	57.1616	Iran
	MVZ 245917	35.9666	56.0684	Iran
	MVZ 248374	36.2688	60.5357	Iran
	MVZ 248376	37.7194	55.9001	Iran
	MVZ 249177	37.7332	55.9006	Iran
	MVZ 249178	37.7332	55.9006	Iran
*Bufotes pseudoraddei*	MVZ 241550	36.5	74.8666	Pakistan
	MVZ 241551	36.5	74.8666	Pakistan
	MVZ 241552	36.5	74.8666	Pakistan
	**MVZ 241553**	35.8833	71.7833	Pakistan
	MVZ 241554	36.0663	72.5166	Pakistan
	MVZ 248375	36.5	74.87	Pakistan
*Bufotes surdus*	MVZ 234217	29.4441	60.5136	Iran
	MVZ 234218	29.4441	60.5136	Iran
	MVZ 234219	29.4441	60.5136	Iran
	**MVZ 234238**	27.8763	60.0955	Iran
	MVZ 234239	28.6066	61.0771	Iran
	MVZ 234240	28.6066	61.0771	Iran
*Bufotes variabilis*	**MVZ 234222**	32.9395	48.2558	Iran
	MVZ 234223	32.9395	48.2558	Iran
	MVZ 234241	31.2133	49.2173	Iran
	MVZ 234242	31.2133	49.2173	Iran
	MVZ 238503	29.615	52.5386	Iran
*Duttaphrynus dhufarensis*	MVZ 241308	26.1503	56.1606	Oman
	**MVZ 242729**	23.0655	57.4701	Oman
	MVZ 242774	17.1001	54.284	Oman
	MVZ 242775	23.0655	57.4701	Oman
*Duttaphrynus himalayanus*	**MVZ 241543**	28.598	83.6469	Himalayas
	MVZ 241544	28.598	83.6469	Himalayas
*Duttaphrynus melanostictus*	MVZ 226298	21.4536	105.6436	Vietnam
	**MVZ 239140**	−3.957	122.5315	Indonesia
*Duttaphrynus olivaceus*	**MVZ 234225**	27.2035	60.6785	Iran
	MVZ 234226	27.1853	60.5895	Iran
	MVZ 234227	27.1853	60.5895	Iran
	MVZ 234228	27.1853	60.5895	Iran
	MVZ 234235	25.2563	60.8326	Iran
	MVZ 234236	25.2563	60.8326	Iran
	MVZ 234237	25.2703	60.7553	Iran
*Duttaphrynus stomaticus*	**MVZ 237424**	34.4366	70.4483	Afghanistan
	MVZ 248377	25.7768	66.6256	Pakistan
	MVZ 248378	25.7768	66.6256	Pakistan
	MVZ 248379	25.7768	66.6256	Pakistan
	MVZ 248380	24.3516	70.7573	Pakistan
	MVZ 248382	24.3516	70.7573	Pakistan
	MVZ 248383	24.3516	70.7573	Pakistan
*Duttaphrynus stuarti*	CAS 242587	27.7746	98.3354	China

Museum numbers in bold represent samples included in the dating analyses. Complete information about gene sampling is included in Additional file 2. Asterisks indicate new name combinations for species.

### DNA extraction and amplification

Whole genomic DNA was extracted from liver samples using a high-salt DNA extraction [46]. We obtained a combination of sequence data from two mitochondrial markers, NADH dehydrogenase subunit 2 (*ND2*) and 16S ribosomal RNA (16S), and partial exonic sequence from the nuclear markers Recombination activating gene 1 (RAG1). The three loci (ND2, 16S, and RAG1) were amplified using the primer pairs 16SA and 16SB [47] for the 16S rRNA partial gene fragment, MET F1 L4437 and TRP R3 [48] for the ND2 partial gene fragment, and the primer pairs MartF1 and AmpR1 [49] were used to amplify RAG1*.*

Polymerase chain reactions (PCRs) were carried out in 12.5 μl volumes consisting of: 1.25 μl Roche 10x (500 mM Tris/HCl, 100 mM KCl, 50 mM (NH_4_)_2_ SO_4_, 20 mM MgCl_2_, pH = 8.3), 0.75 μl 25 mM MgCl_2_, 0.75 μl 2 mM DNTPs, 0.25 μl 10.0 μM forward primer, 0.25 μl 10.0 μM reverse primer, 8.40 μl H_2_O, 0.10 μl Taq, and 0.75 μl DNA. Amplification of both *ND2* and *16S* involved initial denaturation at 94 °C for 4 min, followed by 35 cycles of 95 °C for 60 s, 51 °C for 60 s, 72 °C for 90 s, and a final extension at 72 °C for 7 min. The amplification of RAG1 followed the extended touchdown gradient reported by [41], and involved initial denaturation at 95 °C for 4 min, followed by a first program of 15 cycles of 95 °C for 30 s, 60 °C for 30 s, and 72 °C for 90 s (decreasing annealing temperature by −1 °C per cycle), then a second program consisting of 20 cycles of 95 °C for 30 s, 45 °C for 30 s, and 72 °C for 60 s, with a final extension at 72 °C for 10 min.

The PCR amplifications were visualized on an agarose gel and cleaned using ExoSAP-IT (USB). Gene products were sequenced using BigDye v3.1 on an ABI3730 (Applied Biosystems). Newly generated sequences were edited using Geneious Pro [50]. All newly generated sequences are deposited in GenBank (Accession numbers: KT031406–KT031518 [16S]; KT031519–KT031601 [ND2]; KT031602–KT031708 [RAG-1]).

### GenBank sampling

To provide the most updated bufonid phylogeny for placing our focal taxa, we included GenBank data of representatives of all available unique bufonids (234 species, 39 genera) and several outgroups (seven species) (Additional file 2). The resulting data matrix is largely based on the alignment produced by Pyron and Wiens [39] for their comprehensive analysis of amphibian sequence data (Dryad repository doi:10.5061/dryad.vd0m7). Their matrix was composed of 12 loci: nine nuclear genes consisting of C-X-C chemokine receptor type 4 (CXCR4), histone 3a (H3A), sodium–calcium exchanger (NCX1), pro-opiomelanocortin (POMC), recombination-activating gene 1 (RAG1), rhodopsin (RHOD), seventh-in-absentia (SIA), solute-carrier family 8 (SLC8A3), and tyrosinase (TYR); and three mtDNA loci including cytochrome b (cyt-b), and the large and small sub-units of the mitochondrial ribosome genes (12S/16S; without tRNAs). We conducted GenBank searches for additional taxa not included in this data matrix, and we added sequence data for NADH dehydrogenase subunit 2 (ND2), which has been sequenced for many bufonid taxa. The final data matrix containing both GenBank and newly generated sequence data consists of 243 bufonid lineages and 7 outgroup species. The taxonomy of the family Bufonidae has been under revision, and names of several genera have been changed to reflect evolutionary relationships [34, 35]. Species names were updated accordingly and are here presented using the newest taxonomy from AmphibiaWeb [34] and the Amphibian Species of the World v6.0 [35]. GenBank numbers for all sequence data included are given in Additional file 2.

### Sequence alignment

All protein-coding genes were aligned using MUSCLE [51], and subsequently translated to ensure conservation of reading frame. The 12S and 16S sequences were initially aligned using Clustal Omega [52], manually adjusted by eye, and poorly aligned regions were trimmed from the alignment. Trimmed sequences were then realigned using Clustal Omega, with some slight manual adjustments. The final concatenated alignment consists of 13 loci, 250 taxa, and 10,492 base pairs. The matrix is composed of the following data: 12S, 215 sequences (85 %, 1,011 bp); 16S, 236 sequences (93 %, 1,223 bp); cyt-b, 91 sequences (36 %, 1,122 bp); ND2, 78 sequences (30 %, 1,035 bp); CXCR4, 114 sequences (45 %, 732 bp); H3A, 38 sequences (15 %, 328 bp); NCX1, 60 sequences (23 %, 1,275 bp); POMC, 69 sequences (27 %, 550 bp); RAG1, 101 sequences (40 %, 840 bp); RHOD, 38 sequences (15 %, 315 bp); SIA, 40 sequences (16 %, 397 bp); SLC8A3, 14 sequences (5 %, 1,132 bp); and TYR, 12 sequences (4 %, 532 bp). The mean sequence length is 3,185 bp, and the range in length across taxa is 375 to 7,886 bp. The proportion of missing data across the matrix is approximately 70 %. The full alignment is provided in Additional file 3.

### Phylogenetic analyses

We used PartitionFinder to simultaneously determine our best partitioning strategy and models for each partition subset [53]. The greedy search algorithm was employed, and model selection was conducted using the Bayesian information criterion (BIC). Due to the large size and complexity of the molecular data set, we did not allow for partitioning of genes by codon position. The best partitioning scheme of the full data set includes four gene partitions: 12S and 16S: GTR + G + I; ND2 and cyt-b: GTR + G + I; RHOD, SIA, TYR: HKY + G; and CXCR4, H3A, NCX1, POMC, RAG1, and SLC8A3: GTR + G + I. We conducted Bayesian analyses using MrBayes v3.2 [54, 55], and parallel runs utilizing four MCMC chains were allowed to run for 2×10^7^ million generations, with sampling every 1000 generations. Runs were assessed using Tracer v1.6 [56] to ensure key parameters had reached stationarity (ESS values >150). The first 25 % of the total number of generations were discarded as burn-in and a maximum clade credibility tree was calculated from the remaining trees (30,000) using TreeAnnotator v1.8.1 [57]. We performed maximum likelihood analyses of the partitioned data set using GARLI v2.0 [58]. Using default parameters in the ML search algorithm, 10 replicate searches for the best point estimate topology were conducted, and the tree with the best likelihood was selected as a guide tree for the bootstrap analyses. The Garli Web Service [59], functioning on the Lattice Project grid system [60], was used to execute 1000 nonparametric bootstrap replicates asynchronously in parallel using the default stopping criteria. A maximum clade credibility tree was generated from the 1000 replicates using TreeAnnotator v1.8.1 [57].

### Divergence dating analyses

To infer the timing of lineage divergences of sampled Arabian amphibian taxa, we carried out dating analyses in BEAST v1.8.1 [57]. We included four internal calibration points, following recommendations of [36, 37]:

(A) A minimum age of 20 million years (Myr) for the split between North- and Central America based on the fossil *Bufo praevis* [61]. This was enforced using a lognormal distribution with real space mean of 10, log(stdev) of 1, offset of 19, and initial value of 21, creating the following credibility interval: 5 % = 20.1, 95 % = 50.4.
(B) A minimum age of 18 Mya for the stem origin of toads belonging to the *Bufotes viridis* complex [62, 63]. This was enforced using a lognormal distribution with real space mean of 10, log(stdev) of 1, offset of 17, and initial value of 19, creating the following credibility interval: 5 % = 18.1, 95 % = 48.4.
(C) A minimum age of 11 Myr for the origin of the *Rhinella marina* group (sensu [64]) based on a fossil from the Middle Miocene [65]. This was enforced using a lognormal distribution with real space mean of 10, log(stdev) of 1, offset of 10, and initial value of 12, creating the following credibility interval: 5 % = 11.1, 95 % = 41.4.
(D) A minimum age of 9.6 Myr for the origin of toads belonging to the *Bufo bufo* group based on the appearance of a *Bufo bufo* fossil from the Miocene of Europe [62]. This was enforced using a lognormal distribution with real space mean of 10, log(stdev) of 1, offset of 8, and initial value of 9, creating the following credibility interval: 5 % = 9.1, 95 % = 39.4.

In addition, we used a minimal constraint on the family Bufonidae for the oldest known bufonid fossils recovered from Paleocene deposits in both Brazil and France [66–68]. This was enforced using three different approaches: 1) a lognormal distribution with a real space mean of 15, log(stdev) of 1, offset of 55, and initial value of 65, producing a credibility interval of (5 % = 56.1, 95 % = 102.1); 2) a normal distribution with a mean of 80, a standard deviation of 14, and initial value of 65, creating a credibility interval of (5 % = 56.9, 95 % = 103); and 3) an exponential distribution with a mean of 20, offset of 57, and initial value of 60, producing a credibility interval of (5 % = 58.0, 95 % = 116.9).

Dating analyses were initially run using the full molecular data set of 13 loci and 253 taxa; analyses behaved very poorly and failed to converge even after several hundred million generations. Therefore, the data set was reduced to include only the most well sampled genes: 12S, 16S, ND2, CXCR4, and RAG1. Data were grouped into three partitions: 1) 12S and 16S, 2) ND2, and 3) CXCR4 and RAG1. Inclusion of taxa in the reduced data set required partial sequences for at least two of these three partitions. The final alignment for all subsequent dating analyses included 132 taxa, 5 loci, and 4,840 bp. The average sequence length was 3,051 bp, with a range of 811 to 4,640 bp, and the alignment contained 37 % missing data.

Dating analyses were run for 2×10^7^ generations with sampling every 2000 generations. For all analyses, we used the Yule model of speciation as our tree prior, applied an uncorrelated relaxed lognormal clock, and unlinked clock and substitution models. The partitioning scheme and substitution models are as follows: 12S and 16S: GTR + G + I; ND2: GTR + G + I; CXCR4 and RAG1: GTR + G + I. Runs were assessed using Tracer v1.6 [56] to examine convergence. A burn-in of 25 % was discarded and maximum clade credibility trees were created from a total of 7,500 trees for each analysis.

To explore the effects of calibration choices, we ran multiple analyses with different combinations of calibrations enforced. Fixing the age of the Bufonidae, we explored every permutation of the internal calibrations resulting in 14 sets of analyses (Additional file 4). These 14 analyses revealed that various combinations of calibrations had little overall effect on dating results throughout the tree (Additional file 4). We therefore focused on exploring the effects of the shape of the calibration prior for the age of the Bufonidae in our final dating analyses. This produced three analyses: (A1) all four internal calibrations with a lognormal prior for the Bufonidae, (A2) all four internal calibrations with a normal distribution prior for the Bufonidae, and (A3) all four internal calibrations with an exponential prior for the Bufonidae. Input xml files of these analyses and resulting consensus trees are available from the Dryad Digital Repository: http://dx.doi.org/10.5061/dryad.bc578 [69].

## Additional files

Additional file 1:
**Phylogeny of the Bufonidae.** A complete full-length version of Fig. 2. Additional file 2:
**Taxon Sampling.** Voucher, locality information, and gene sampling of new specimens, and GenBank numbers of published data. Additional file 3:
**Final Alignment.** Full nexus alignment of 13 loci and 250 taxa, and relevant data partitions. Additional file 4:
**Internal Calibration Dating Results.** Dating estimates for nodes of interest under all permutations of the four internal calibrations. Additional file 5:
**Labeled Chronogram.** The chronogram resulting from dating analysis A1 (lognormal prior on Bufonidae) with calibrated nodes highlighted and all nodes numbered. Additional file 6:
**Dating Comparisons Across Chronogram Nodes.** A supporting table with all numbered node age estimates and intervals.
