# Supplementary figures and images for: Historical biogeography resolves the origins of endemic Arabian toad lineages (Anura: Bufonidae): Evidence for ancient vicariance and dispersal events with the Horn of Africa and South Asia
Source: BMC Evol Biol. 2015 Aug 6;15:152. doi: 10.1186/s12862-015-0417-y (PMC4527211; doi:10.1186/s12862-015-0417-y)

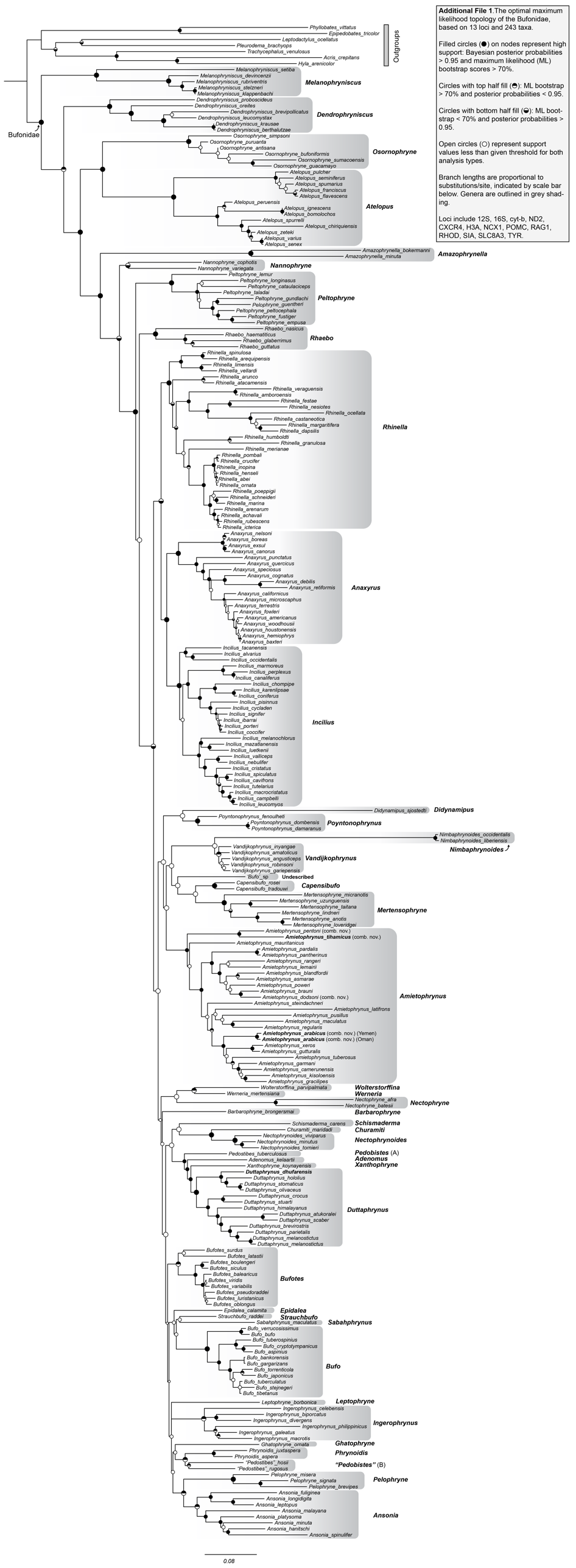

Supplement: Additional file 1: — Phylogeny of the Bufonidae. A complete full-length version of Fig. 2. [file 12862_2015_417_MOESM1_ESM.tiff]

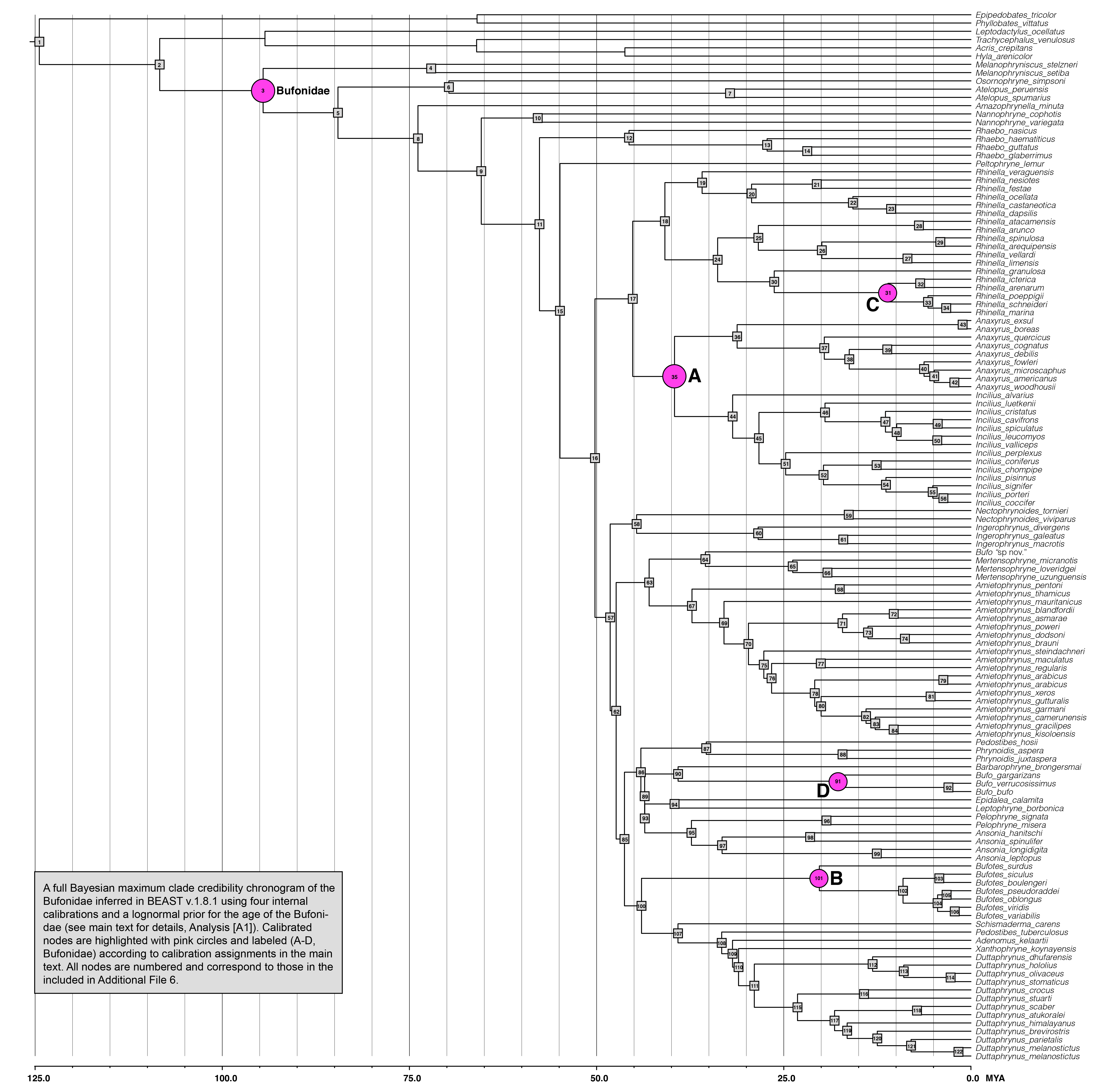

Supplement: Additional file 5: — Labeled Chronogram. The chronogram resulting from dating analysis A1 (lognormal prior on Bufonidae) with calibrated nodes highlighted and all nodes numbered. [file 12862_2015_417_MOESM5_ESM.tif]
