# Supplementary material for: Historical biogeography resolves the origins of endemic Arabian toad lineages (Anura: Bufonidae): Evidence for ancient vicariance and dispersal events with the Horn of Africa and South Asia
Source: BMC Evol Biol. 2015 Aug 6;15:152. doi: 10.1186/s12862-015-0417-y (PMC4527211; doi:10.1186/s12862-015-0417-y)
Supplement: Additional file 6: — Dating Comparisons Across Chronogram Nodes. A supporting table with all numbered node age estimates and intervals. [file 12862_2015_417_MOESM6_ESM.docx]

Additional File 6 Table. Summary of divergence dates for all nodes across all three dating analyses.

| Label | Node | Bufonidae Lognormal Prior  (A1) | | Bufonidae Normal Prior  (A2) | | Bufonidae Exponential Prior  (A3) | |
| --- | --- | --- | --- | --- | --- | --- | --- |
|  |  | Median  Age (Ma) | 95% HPD | Median  Age (Ma) | 95% HPD | Median  Age (Ma) | 95% HPD |
|  | 1 | 124.8 | [84.1–177.1] | 121.5 | [89.3–160.3] | 128.0 | [85.7–182.1] |
|  | 2 | 108.7 | [76.7–152.2] | 108.2 | [84.5–135.0] | 115.3 | [79.8–155.6] |
| **Bufonidae Calibration** | **3** | **94.9** | **[64.6–129.8]** | **93.5** | **[73.0–114.0]** | **100.3** | **[70.1–133.1]** |
|  | 4 | 72.4 | [44.6–105.7] | 71.2 | [44.0–97.1] | 77.0 | [50.2–110.8] |
|  | 5 | 84.9 | [58.4–116.4] | 83.7 | [65.6–103.0] | 89.7 | [62.3–117.7] |
|  | 6 | 70.1 | [43.9–99.3] | 69.7 | [48.6–90.8] | 74.5 | [49.1–102.3] |
|  | 7 | 32.5 | [16.7–55.0] | 31.9 | [17.6–46.8] | 34.8 | [17.8–52.9] |
|  | 8 | 74.2 | [50.6–101.0] | 73.1 | [56.6–90.4] | 78.7 | [52.9–102.9] |
|  | 9 | 65.8 | [45.8–90.0] | 64.6 | [49.7–79.7] | 69.4 | [48.9–92.0] |
|  | 10 | 58.2 | [36.3–80.6] | 57.2 | [41.1–74.5] | 62.2 | [40.3–85.8] |
|  | 11 | 65.5 | [40.4–78.5] | 56.7 | [44.4–71.1] | 60.5 | [42.9–79.2] |
|  | 12 | 46.0 | [29.1–65.7] | 44.3 | [32.9–59.3] | 47.4 | [31.5–65.7] |
|  | 13 | 27.6 | [15.5–42.6] | 26.3 | [16.6–36.8] | 28.0 | [16.5–41.2] |
|  | 14 | 22.2 | [11.7–35.7] | 21.3 | [11.9–30.7] | 22.6 | [11.1–34.3] |
|  | 15 | 55.2 | [38.8–75.8] | 53.8 | [42.5–68.0] | 57.0 | [39.7–73.7] |
|  | 16 | 50.6 | [36.2–69.3] | 49.2 | [39.0–61.5] | 51.9 | [37.3–67.7] |
|  | 17 | 45.5 | [31.8–62.3] | 44.4 | [34.6–55.9] | 46.9 | [33.4–60.9] |
|  | 18 | 41.2 | [28.8–56.8] | 40.1 | [30.7–50.7] | 42.2 | [29.2–55.0] |
|  | 19 | 36.3 | [24.9–50.2] | 34.7 | [26.3–45.0] | 36.8 | [24.5–48.8] |
|  | 20 | 29.6 | [19.5–41.1] | 27.6 | [19.9–37.0] | 29.8 | [20.1–40.3] |
|  | 21 | 20.9 | [11.9–29.7] | 19.1 | [12.2–27.9] | 21.4 | [12.7–30.5] |
|  | 22 | 16.1 | [8.8–23.6] | 15.5 | [9.4–21.3] | 17.2 | [9.9–24.5] |
|  | 23 | 11.0 | [5.5–17.3] | 10.6 | [5.8–16.3] | 12.0 | [5.8–17.8] |
|  | 24 | 34.2 | [23.3–48.2] | 33.6 | [24.6–43.8] | 35.2 | [23.9–46.6] |
|  | 25 | 28.7 | [18.7–41.7] | --- | --- | --- | --- |
|  | 26 | 20.2 | [11.7–30.0] | 19.5 | [11.5–28.8] | 20.9 | [11.1–29.8] |
|  | 27 | 8.8 | [3.7–14.5] | 8.3 | [4.0–14.2] | 8.9 | [3.1–14.8] |
|  | 28 | 7.3 | [3.3–12.6] | 6.7 | [3.2–11.9] | 7.6 | [2.9–12.5] |
|  | 29 | 4.4 | [1.6–7.5] | 3.6 | [1.7–6.6] | 4.8 | [1.5–7.4] |
|  | 30 | 26.6 | [17.5–38.2] | 26.5 | [18.0–36.5] | 27.7 | [17.7–37.8] |
| **Calibration C** | **31** | **11.4** | **[10.1–12.8]** | **11.0** | **[10.1–12.5]** | **11.9** | **[10.1–12.9]** |
|  | 32 | 7.1 | [3.5–10.0] | 6.6 | [3.3–9.9] | 7.8 | [3.7–9.9] |
|  | 33 | 6.1 | [2.8–8.9] | 5.6 | [3.1–8.7] | 6.6 | [3.1–9.2] |
|  | 34 | 3.6 | [1.5–5.8] | 3.3 | [1.4–5.7] | 4.2 | [1.4–6.0] |
| **Calibration A** | **35** | **39.9** | **[27.1–54.7]** | **38.9** | **[30.4–50.6]** | **41.3** | **[28.8–53.9]** |
|  | 36 | 31.6 | [21.1–44.6] | 31.0 | [22.5–41.6] | 32.6 | [22.0–45.0] |
|  | 37 | 19.9 | [12.8–29.2] | 19.9 | [13.1–27.5] | 20.8 | [13.2–29.5] |
|  | 38 | 16.6 | [10.6–24.5] | 16.7 | [10.6–23.2] | 17.5 | [10.7–24.7] |
|  | 39 | 11.5 | [6.2–18.1] | 11.3 | [6.3–17.2] | 12.5 | [6.7–18.7] |
|  | 40 | 6.6 | [3.5–10.2] | 6.5 | [3.9–10.2] | 7.5 | [3.5–10.7] |
|  | 41 | 5.2 | [2.6–8.2] | 5.2 | [2.9–8.3] | 6.1 | [2.7–8.4] |
|  | 42 | 2.5 | [0.8–4.2] | 2.2 | [0.9–4.0] | 3.1 | [0.7–4.2] |
|  | 43 | 1.4 | [0.4–2.3] | 1.0 | [0.4–2.1] | 2.0 | [0.4–2.3] |
|  | 44 | 32.2 | [21.0–44.8] | 31.4 | [23.2–41.0] | 33.1 | [22.7–45.3] |
|  | 45 | 28.7 | [19.3–40.4] | 27.9 | [20.3–36.2] | 29.7 | [19.3–39.4] |
|  | 46 | 19.8 | [12.6–28.5] | 19.0 | [13.0–25.9] | 20.6 | [12.8–28.7] |
|  | 47 | 11.8 | [7.1–16.8] | 11.0 | [7.5–15.9] | 12.7 | [7.6–17.0] |
|  | 48 | 10.3 | [6–14.7] | 9.6 | [6.2–13.4] | 11.2 | [6.8–14.9] |
|  | 49 | 4.8 | [2.3–7.4] | 4.5 | [2.7–7.2] | 5.3 | [2.4–7.4] |
|  | 50 | 4.8 | [2.3–7.3] | 4.4 | [2.4–6.9] | 5.7 | [2.5–8.1] |
|  | 51 | 25.1 | [16.3–35.0] | 24.4 | [17.3–32.0] | 26.0 | [17.1–35.3] |
|  | 52 | 20.0 | [12.7–29.2] | 19.5 | [13.5–26.0] | 20.9 | [12.8–28.2] |
|  | 53 | 12.9 | [6.9–19.2] | 12.1 | [7.5–17.0] | 13.6 | [7.0–19.1] |
|  | 54 | 11.7 | [6.8–17.8] | 11.6 | [7.3–16.8] | 12.6 | [7.1–17.9] |
|  | 55 | 5.4 | [2.7–8.2] | 5.0 | [2.9–7.7] | 6.0 | [3.0–8.0] |
|  | 56 | 4.0 | [1.9–6.2] | 3.6 | [1.7–5.9] | 4.5 | [2.0–6.4] |
|  | 57 | 48.5 | [34.5–66.4] | 47.3 | [36.8–58.2] | 49.7 | [34.8–64.3] |
|  | 58 | 45.0 | [30.5–61.8] | 43.9 | [31.9–55.0] | 47.1 | [32.0–62.5] |
|  | 59 | 16.6 | [8.0–27.9] | 15.8 | [8.2–24.7] | 17.3 | [8.3–26.2] |
|  | 60 | 28.8 | [16.5–41.1] | 27.4 | [17.2–38.4] | 29.2 | [18.0–41.2] |
|  | 61 | 17.4 | [7.9–27.6] | 16.2 | [8.5–25.3] | 17.3 | [8.6–27.3] |
|  | 62 | 47.7 | [33.1–65.1] | 46.6 | [36.5–57.8] | 49.7 | [36.8–66.3] |
|  | 63 | 43.3 | [29.8–60.1] | 42.6 | [33.3–52.7] | 44.1 | [31.5–58.9] |
|  | 64 | 35.8 | [23.1–52.0] | 34.9 | [24.5–46.8] | 35.9 | [22.5–51.4] |
|  | 65 | 24.1 | [14.2–34.5] | 22.7 | [15.0–32.4] | 23.3 | [14.1–34.6] |
|  | 66 | 19.5 | [10.2–29.8] | 18.4 | [11.2–27.5] | 18.8 | [10.0–29.5] |
| **TMRCA: *Amietophrynus*** | **67** | **37.6** | **[25.6–51.5]** | **36.5** | **[28.1–45.5]** | **37.2** | **[27.1–50.4]** |
| **TMRCA: *A. tihamicus*** | **68** | **17.9** | **[9.0–26.6]** | **16.8** | **[9.2–25.4]** | **17.0** | **[9.3–25.9]** |
|  | 69 | 33.3 | [22.5–45.5] | 32.4 | [25.9–40.6] | 32.9 | [23.9–44.7] |
|  | 70 | 30.0 | [20.5–41.5] | 29.3 | [22.6–36.9] | 29.7 | [21.4–40.0] |
|  | 71 | 17.5 | [10.7–25.2] | 16.9 | [11.6–22.4] | 17.1 | [11.4–24.9] |
|  | 72 | 10.6 | [5.1–16.9] | 10.2 | [5.4–16.1] | 10.4 | [5.0–16.1] |
|  | 73 | 14.1 | [8.0–20.7] | 13.5 | [8.9–18.9] | 13.7 | [8.4–20.5] |
|  | 74 | 9.2 | [3.7–15.0] | 8.7 | [4.1–13.9] | 8.9 | [4.2–14.9] |
|  | 75 | 28.0 | [18.4–38.3] | 27.3 | [20.4–34.0] | 27.7 | [19.6–37.2] |
|  | 76 | 26.9 | [17.2–37.5] | 26.4 | [19.9–34.0] | 26.4 | [18.5–35.9] |
|  | 77 | 20.4 | [11.7–28.8] | 20.1 | [13.5–26.8] | 19.5 | [12.6–27.8] |
| **TMRCA: *A. arabicus*** | **78** | **21.2** | **[14.2–29.2]** | **20.5** | **[15.1–26.3]** | **20.7** | **[14.8–29.2]** |
|  | 79 | 4.0 | [1.5–6.7] | 3.5 | [1.6–6.1] | 3.5 | [1.5–7.3] |
|  | 80 | 20.4 | [13.6–27.7] | 19.7 | [14.1–25.0] | 19.8 | [13.5–27.9] |
|  | 81 | 5.7 | [2.6–9.6] | 5.2 | [2.4–9.0] | 5.2 | [2.4–9.2] |
|  | 82 | 14.3 | [8.5–20.4] | 13.4 | [9.0–18.6] | 13.6 | [8.4–20.5] |
|  | 83 | 13.1 | [7.9–18.3] | 11.4 | [7.4–16.3] | 11.5 | [6.4–17.4] |
|  | 84 | 10.6 | [5.7–15.3] | 10.0 | [5.8–14.5] | 9.9 | [5.4–15.5] |
|  | 85 | 46.6 | [30.9–61.0] | 44.4 | [34.6–55.0] | 46.9 | [33.7–62.3] |
|  | 86 | 44.4 | [31.4–61.4] | 42.2 | [32.2–52.3] | 44.9 | [32.4–60.8] |
|  | 87 | 35.7 | [23.1–51.1] | 34.7 | [23.8–45.6] | 35.5 | [23.9–50.1] |
|  | 88 | 17.5 | [8.3–28.2] | 16.2 | [7.8–26.0] | 17.0 | [8.9–27.6] |
|  | 89 | 43.9 | [30.6–60.5] | --- | --- | --- | --- |
|  | 90 | 39.4 | [28.0–56.8] | --- | --- | --- | --- |
| **Calibration D** | **91** | **18.1** | **[9.9–27.7]** | **16.8** | **[10.6–24.7]** | **18.2** | **[10.9–28.6]** |
|  | 92 | 3.3 | [1.3–5.5] | 2.9 | [1.4–5.0] | 3.0 | [1.3–5.5] |
|  | 93 | 43.9 | [30.3–61.1] | --- | --- | --- | --- |
|  | 94 | 39.5 | [27.4–57.4] | --- | --- | --- | --- |
|  | 95 | 37.3 | [25.2–51.4] | 36.1 | [27.0–45.4] | 38.0 | [26.8–50.7] |
|  | 96 | 19.3 | [11.3–29.7] | 19.0 | [12.0–26.3] | 20.3 | [11.2–30.4] |
|  | 97 | 33.2 | [22.0–46.2] | 32.4 | [23.4–41.7] | 33.8 | [22.9–45.8] |
|  | 98 | 21.5 | [12.8–32.7] | 21.5 | [13.4–30.5] | 22.1 | [13.2–32.3] |
|  | 99 | 12.5 | [5.9–21.4] | 12.2 | [5.5–19.8] | 12.6 | [6.0–20.9] |
|  | 100 | 44.3 | [30.5–59.4] | 42.6 | [32.9–52.6] | 45.6 | [32.4–60.8] |
| **Calibration B** | **101** | **20.6** | **[17.3–27]** | **20.6** | **[17.1–29.5]** | **20.4** | **[17.1–28.0]** |
|  | 102 | 9.4 | [5.1–13.9] | 8.9 | [5.4–13.2] | 9.0 | [4.9–13.7] |
|  | 103 | 4.6 | [1.8–7.4] | 4.0 | [1.8–6.9] | 4.2 | [1.8–7.5] |
|  | 104 | 4.8 | [2.3–7.1] | 4.6 | [2.5–7.0] | 4.4 | [2.4–7.5] |
|  | 105 | 3.6 | [1.5–5.4] | 3.4 | [1.7–5.3] | 3.2 | [1.4–5.5] |
|  | 106 | 2.5 | [0.2–4.7] | 2.1 | [0.2–4.5] | 2.2 | [0.3–4.6] |
|  | 107 | 39.5 | [26.2–54.0] | 37.8 | [28.1–47.3] | 39.1 | [25.8–52.8] |
|  | 108 | 33.6 | [22.7–47.0] | 32.3 | [24.0–40.6] | 33.7 | [23.0–45.5] |
|  | 109 | 32.2 | [21.2–44.4] | 31.1 | [23.1–39.3] | --- | --- |
|  | 110 | 31.4 | [20.7–43.0] | 29.9 | [22.9–38.1] | 31.3 | [21.0–42.1] |
| **TMRCA: *Duttaphrynus*** | **111** | **29.3** | **[19.3–40.5]** | **27.8** | **[20.9–35.1]** | **29.2** | **[20.4–40.0]** |
| **TMRCA: *D. dhufarensis*** | **112** | **13.5** | **[7.9–20.9]** | **12.9** | **[8.2–18.6]** | **13.5** | **[8.4–20.1]** |
|  | 113 | 9.3 | [4.5–14.8] | 8.6 | [4.8–13.4] | 9.2 | [5.0–14.6] |
|  | 114 | 3.0 | [1.0–5.1] | 2.6 | [1.1–4.7] | 2.7 | [1.0–5.2] |
|  | 115 | 23.5 | [15.1–32.7] | 22.4 | [16.5–28.9] | 23.4 | [15.9–32.3] |
|  | 116 | 14.6 | [7.7–22.3] | 13.8 | [8.2–20.9] | 14.4 | [8.2–22.2] |
|  | 117 | 18.6 | [11.1–25.4] | 17.5 | [12.7–22.9] | 18.3 | [12.3–25.7] |
|  | 118 | 7.5 | [3.4–11.7] | 6.9 | [3.6–10.8] | 7.3 | [3.6–11.7] |
|  | 119 | 16.8 | [9.9–23.2] | 15.9 | [11.1–21.1] | 16.7 | [11.0–23.5] |
|  | 120 | 12.8 | [7.3–18.5] | 12.0 | [7.7–17.0] | 12.7 | [7.9–18.4] |
|  | 121 | 8.3 | [4.2–13.0] | 7.9 | [4.5–12.2] | 8.2 | [4.4–12.7] |
|  | 122 | 2.0 | [0.1–4.4] | 1.6 | [0.2–4.2] | 1.7 | [0.1–4.2] |

The Table summarizes median age estimates and associated 95% HPD intervals for each node and across each of the three dating analyses (A1, A2, A3). Discrepancies between tree topologies (conflicting nodes) are represented with dashes. Several key nodes are labeled for quick reference.
